# A Case of Abscess of the Uterine Appendages in the Non-Puerperal State

**Published:** 1855-09

**Authors:** F. M. McCabe

**Affiliations:** Resident Graduate at the Louisville Marine Hospital


### Art. III.—A CASE OF ABSCESS OF THE UTERINE APPENDAGES IN THE NON-PUERPERAL STATE.

BY F. M. M'CABE, M. D., RESIDENT GRADUATE AT THE LOUISVILLE MARINE HOSPITAL.

The annexed case is reported not because it is supposed to exhibit anything new, or even to illustrate, in a remarkable manner, the symptoms of non-puerperal abscess, but simply from a conviction of the liability of mistaking the disease for other more commonly recognized uterine lesions, with which it shows, to a great extent, the ordinary congeries of "uterine symptoms."

Bridget Grffin, aged thirty-three, a native of Ireland, was admitted to Louisville Marine Hospital Aug. 2, 1855, for supposed prolapsus uteri, of which affection her physician had assured her she was the subject. Her menstrual functions had been uniformly healthy up to the present attack. Within twelve months had had intermittent fever irregularly, from which, however, she had been entirely free for two months prior to entry. She was attacked on July 22d by chilly sensations, severe lumbar and hypogastric pains, a sensation of weight deeply seated in the pelvis upon attempting to walk, with much vesical irritation, as a consequence of protracted exposure to toil while washing clothes, being then on the eve of menstruation, which indeed ensued on the following day, but stopped abruptly after lasting less than twenty-four hours, (her menstrual period usually occupying three days and nights.) On the morning of the 23d she had a severe chill, followed by high febrile reaction, chills continued to recur at about the same hour for six successive days, but growing with each paroxysm progressively lighter, until after the sixth return, when they spontaneously ceased. At this time the lumbar and hypogastric pains were much lessened in intensity. On the tenth day from the attack the notice of the nurse was suddenly called to a profuse purulent discharge from the vagina, which amounted perhaps to a quart. The patient was immediately placed upon a vessel and states that there was a further discharge of about a pint of bloody pus. A continuous but small discharge existed up to the time of her entry into the Hospital.

*Condition at the time of Admission.*—August 3d: No purulent discharge from vagina. On examination with the speculum a small perforation of the left vaginal wall is seen; uterus *in situ* and of healthy aspect. The finger introduced into the vagina encounters an indurated swelling encroaching upon the cul de sac of this canal; an indurated swelling surrounding the aperture. Patient complains of no pain. Pressure over the abdomen is accompanied by tenderness which is most marked over the left iliac region, where she expressed herself as having suffered most from pain in the beginning of her attack. The indications for medication not being very urgent I contented myself by directing fomentations to be applied to the abdomen.

August 5th: Patient has a chill; and on inquiry found that she had had one yesterday. August 6th: Ordered quinia grs. ij. every two hours till grs. xxv were taken, with but the effect of retarding the paroxysm several hours. August 7th: Quinia given to cinchonism. August 8th: Cinchonism; continued to escape chill till 13th, when it became necessary to resume the quinine, to which was now added FOWLER's solution The general evidences of anemia being present the patient was also directed to use some of the ferruginous preparations. August 14th: Specular examination shows the point of ulceration to be granulating healthily. August 20th: Menstruous flow re-established, perfectly normal in character. August 25th: Patient left the Hospital cured.

